# Temporal muscle thickness as a feasible sarcopenia marker and outcome predictor after aneurysmal subarachnoid hemorrhage

**DOI:** 10.1007/s00701-025-06562-z

**Published:** 2025-05-29

**Authors:** E. Mohajerani, M. Gümüs, T. F. Dinger, C. Rieß, L. Rauschenbach, J. Rodemerk, C. D. Ziegenfuß, M. Darkwah Oppong, Y. Ahmadipour, Y. Li, P. Dammann, U. Sure, R. Jabbarli

**Affiliations:** 1 Department of Neurosurgery and Spine Surgery, University Hospital Essen, 45147 Essen, Germany; 2 Institute of Diagnostic and Interventional Radiology and Neuroradiology, University Hospital Essen, 45147 Essen, Germany

**Keywords:** Subarachnoid Hemorrhage, Outcome prediction, Sarcopenia, Temporal Muscle Thickness, Prognostic marker

## Abstract

**Background and Purpose:**

Sarcopenia has already been investigated as a prognostic marker for many different cancerous and non-cancerous diseases to prognosticate the clinical course. While being a sarcopenia marker, temporal muscle thickness (TMT) has gained increasing interest in recent years as a potential outcome predictor. The aim of this retrospective study was to investigate the association between TMT and the neurological outcome of patients with aneurysmal subarachnoid hemorrhage (aSAH).

**Methods:**

A retrospective database consisting of consecutive aSAH cases treated from 01/2003 to 06/2016 was used. The initial computed tomography examinations were used to calculate the mean TMT values. Our primary endpoint was unfavorable outcome at 6 months defined as modified Rankin Scale > 3. Secondary endpoints included the occurrence of angiographic vasospasm and intracranial hypertension (> 20mmHg) during aSAH, in-hospital mortality and development of cerebral infarcts. Univariable analyses were conducted and multivariable analyses were performed on significant findings.

**Results:**

The mean TMT value of the final cohort (n = 945) was 7.49mm ± 1.68mm. Of the baseline characteristics, a significant relationship with TMT mean value was found for age (p < 0.0001), sex (p < 0.0001), obesity (p = 0.001), hypothyroidism (p = 0.001), and hyperuricemia (p = 0.026). In the final multivariable analysis, the following study endpoints were independently associated with mTMT: in-hospital mortality (p = 0.035, adjusted odds ratio [aOR] 0.86 per-mm-increase, 95% confidence interval [CI] 0.75–0.99), unfavorable outcome at 6 months (p = 0.018, aOR 0.86, 95% CI 0.76–0.98), intracranial hypertension (p = 0.002, aOR 1.17, 95% CI 1.06–1.29) and the occurrence of angiographic vasospasm (p = 0.011, aOR 0.87, 95% CI 0.78–0.97).

**Conclusions:**

In this study, we found significant correlations between mTMT and the clinical course and outcome of patients with aSAH. Further studies in different patient populations are needed to validate the clinical relevance and prognostic value of TMT for aSAH patients.

**Supplementary Information:**

The online version contains supplementary material available at 10.1007/s00701-025-06562-z.

## Introduction

Subarachnoid hemorrhage (SAH) due to aneurysm rupture is known for some early and late complications, which can significantly influence the further course of the disease [15]. Common outcome predictors include such baseline parameters as the World Federation of Neurosurgical Societies (WFNS) [40, 33], Hunt & Hess [14], and Fisher [9] scales, pupil status [39], the presence of intracerebral hematoma [39, 4], the patients' age [26], early brain edema [37], and aneurysm rebleeding [32, 42]. Further research into various predictors can contribute to construction of accurate prognostication tools and development of individualized treatment regimens aiming the prevention of secondary complications and improvement of patient outcomes. Hence, elaboration of novel predictors not or poorly investigated in the context of aneurysmal SAH (aSAH) is of particular clinical interest.

Sarcopenia, as a state of muscular loss has already been discussed in the literature as a negative predictor of the clinical course and outcome of various morbid conditions [41], including the pancreatic [27] and colorectal cancer [23], as well as to other gastrointestinal tumorous and non-tumorous diseases [31, 25].

The temporal muscle thickness (TMT), as a parameter easily accessible upon a native cranial computed tomography (CT) scan, has been reported to correlate with the skeletal muscle mass and the nutritional status of patients [21]. Therefore, TMT was suggested as a reliable marker of sarcopenia [34], and has been evaluated as a prognostic factor for various neurological diseases like ischemic stroke [1, 24] and glioblastoma [12, 47, 36]. Regarding aSAH and TMT, several studies have been published in recent years that have emphasized the predictive value of TMT for the clinical course and outcome of aSAH patients. However, these studies were mostly limited to the populations from the Asian region and based on a relatively small number of patients [19, 18]. Moreover, the clinical background of the eventual association between sarcopenia and aSAH outcome, and particularly the complications pattern after aSAH related to sarcopenia presence, remain unclear.

This study aimed to determine the value of TMT as a radiographic marker of sarcopenia for the prediction of course and outcome after aSAH.

## Methods

### Patient population

This retrospective study included all consecutive cases with aSAH admitted within 48 h after ictus, had an available pre-treatment CT scan for TMT evaluation, and were treated in our hospital from January 2003 to June 2016.

### aSAH management

All patients were initially admitted to our neurosurgical intensive care unit. After the identification of the bleeding source by digital subtraction angiography (DSA), the ruptured aneurysm was treated within 24 h after admission, either endovascularly or by microsurgical clipping. The post-interventional intensive care management has already been described in our previous papers [6, 5]. In short, the conservative treatment regimen after aneurysm securing consisted of maintaining a mean arterial pressure (MAP) > 70 mmHg, normovolemia and normothermia, as well as oral administration of nimodipine for the first 21 days after the bleeding event. Furthermore, vasospasm monitoring was performed by means of daily transcranial Doppler (TCD) sonographic examinations. In unconscious patients with increase of flow velocities > 120 cm/s in two consecutive days, and in all cases with neurological worsening attributable to cerebral vasospasm [3, 16], a new DSA was performed to confirm and treat the vasospasm with intra-arterial nimodipine and, in selected cases, angioplasty. Acute hydrocephalus was treated by placing an external ventricular drainage (EVD), with implantation of a ventriculoperitoneal shunt (VPS) in patients developing chronic hydrocephalus. Patients underwent regular intracranial pressure (ICP) monitoring via EVD. In case of sustained ICP increase > 20 mmHg, conservative ICP treatment was initiated as previously described [22]. ICP increases refractory to conservative management were treated with decompressive craniectomy (DC). Repeated CT imaging of the neurocranium was performed as part of the evaluation in the context of EVD weaning, within 24 h after every surgical treatment or when clinically necessary.

### Data management

**Fig. 1:**
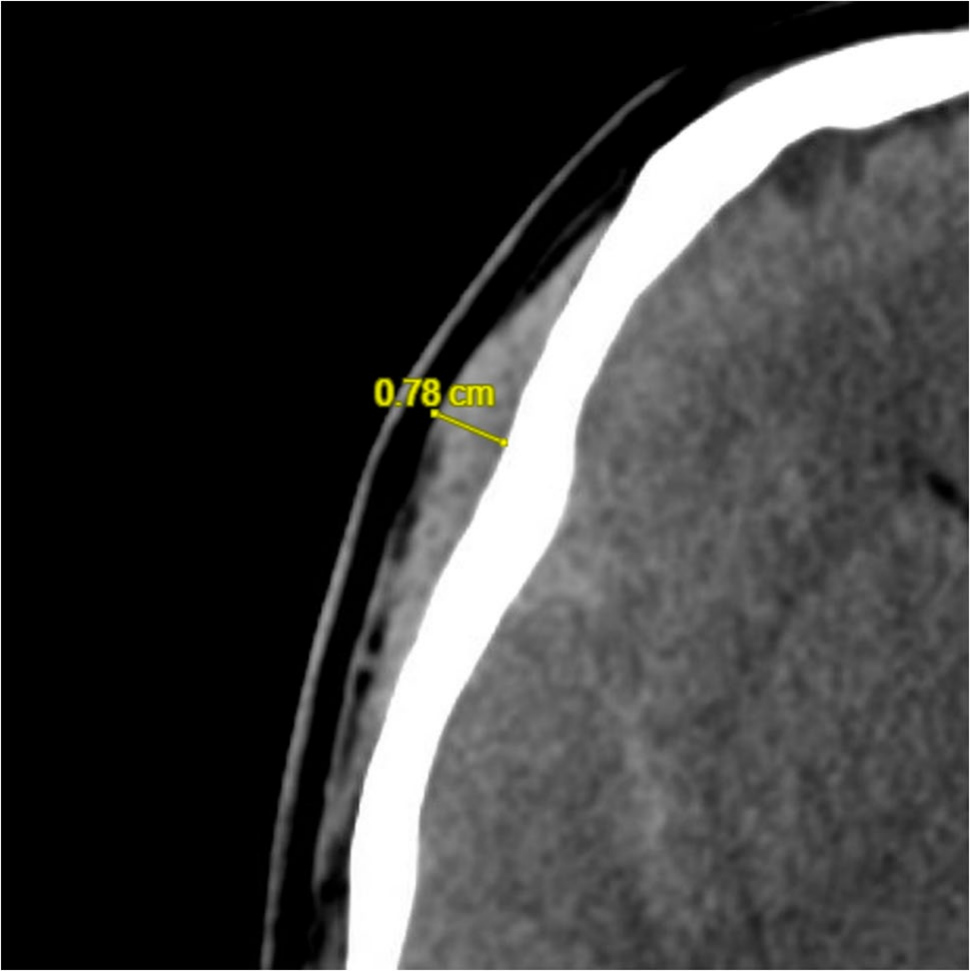
Measurement of the temporal muscle thickness 5 mm above the orbital roof

For TMT measurements, pre-treatment CT scans were analyzed by an evaluator (E.M.) blinded at that moment to the clinical data. The thickness of the temporalis muscle was measured in the axial slice located at 5 mm above the orbit with the tilt of the CT scan being parallel to the skull base. The temporalis muscle was measured three times in succession at its thickest point on both sides of the cranial calvaria. For statistical analysis, the mean value of the TMT (mTMT) was calculated based on the values on both sides (Fig. 1). To assess intra-rater reliability, we used an intra-class correlation coefficient (ICC) based on a two-way mixed model for consistency (ICC(3,k)). For the left side, we determined an ICC of 0.987 for the individual measurements and 0.996 for the mean of the measurements. For the right-hand side, the ICC was 0.964 (individual measurements) and 0.988 (mean of the measurements). These results indicate a high measurement reliability regardless of the side. Data on the demographic, clinical, and radiographic characteristics of patients, including the previous medical history (comorbidities) was collected from the institutional aSAH database. The clinical severity of aSAH was rated using the modified Herniation World Federation of Neurosurgical Societies (hWFNS) [33] scale. The radiographic severity was determined using original Fisher scale [9] dichotomized in low (Fisher 1–2) and high (Fisher 3–4) severity. The following adverse events occurring at the beginning of aSAH or during the intensive care treatment were also recorded for statistical analyses: aneurysm rebleeding, occurrence of cerebral vasospasms confirmed and treated with repetitive DSA, persistent ICP increase > 20 mmHg needing conservative treatment and/or DC, epileptic seizures, new cerebral infarction(s) in follow-up CT scans, and acute coronary syndrome (ACS) as previously described [28]. Moreover, the occurrence of systemic infections was also assessed. Patients suffering from a severe systemic infection within the first two weeks after ictus were categorized as “septic”, provided that they fulfilled the criteria of the quick Sepsis-related Organ Failure Assessment (qSOFA) [10], the other infectious patients not fulfilling qSOFA criteria were documented as “any systemic infections”. The functional outcome at 6-month follow-up was assessed using the modified Rankin Scale (mRS) [44]. Unfavorable outcome was defined as a mRS > 3.

### Study endpoints and statistical analyses

The aim of this study was to investigate the predictive influence of the surrogate marker TMT on the outcome and clinical course of aSAH patients. Our primary study endpoint was defined as unfavorable outcome at 6 months. Secondary endpoints included the association between mTMT with patients’ baseline characteristics, in-hospital mortality, cerebral infarction and adverse events during aSAH, which were divided into:

- cerebral complications: aneurysm rebleeding, angiographic vasospasm, ICP increase > 20 mmHg requiring conservative treatment, DC for ICP increase, shunt dependency, and epilepsy,
- and non-cerebral complications: systemic infections, sepsis, ACS, and thromboembolic events.

In cases of dichotomous endpoints, the associations between mTMT and these endpoints were first tested in univariable analysis using the unpaired t-test or Mann Whitney U-test for normally and non-normally distributed data respectively. For continuous endpoints, Pearson or Spearman correlations were used, as appropriate. The significant results from the univariable analysis were then included into a multivariable analysis to determine independent correlations. In the multivariable analysis for the predictors of adverse events, the associations were adjusted for initial clinical (hWFNS scale) and radiographic (Fisher scale) aSAH severity, presence of acute hydrocephalus, and baseline characteristics associated with mTMT: age, sex, ethnicity, obesity, hypothyroidism, and hyperuricemia. For the multivariable analysis regarding the primary endpoint, the prediction model was enhanced with mTMT-related adverse events (ICP increase and angiographic vasospasm). A p-value < 0.05 was considered statistically significant. Missing data were substituted using multiple imputations. The data was analyzed using SPSS (version 25, SPSS Inc., IBM Company, Chicago, IL, USA) and R (version 4.4.1, R Core Team 2021) with additional R-Studio software for visualization (R-Studio version 2024.12.0,"Kousa Dogwood"Release, https://rstudio.com/products/rstudio/download/).

To assess possible collinearity between the predictors, we examined the correlation between age and mTMT in the model. We found a moderate negative correlation between age and mTMT (Pearson r = −0.239), which does not indicate problematic collinearity. Furthermore, the variance inflation factor (VIF) for all included variables, including mTMT and age, was well below the critical threshold [8] (all VIFs < 1.3). In this respect, multicollinearity was ruled out as a relevant factor influencing our analyses or conclusions.

## Results

### Population characteristics and descriptive data

After excluding non-eligible cases due to late admission and/or absence of the pre-treatment CT scan (n = 50), 945 aSAH patients were included in the final analysis. The mean age of the cohort was 55 years. The proportion of female patients in this cohort was greater than that of males, with 67% of the total population being female. A further detailed list of the baseline characteristics and outcome parameters is shown in Table 1.

**Table 1:** Baseline characteristics, adverse events and outcome data on the final aSAH cohort included in the analysis

Parameter	Mean ± SD or Count (%*)
Age, years	55 (± 14)
Female sex	635 (67.2%)
Ethnicity (Caucasian)	902 (95.4%)
hWFNS scale Grade I Grade II Grade III Grade IV Grade V	159 (16.8%) 229 (24.2%) 149 (15.8%) 327 (34.6%) 81 (8.6%)
Fisher scale Grade 1 Grade 2 Grade 3 Grade 4	23 (2.6%) 99 (11.4%) 197 (22.6%) 551 (63.3%)
Acute hydrocephalus	683 (72.3%)
ICP increase requiring medical treatment	435 (46.5%)
Angiographic vasospasm	230 (24.3%)
Cerebral infarction	469 (49.9%)
In-hospital mortality	177 (18.7%)
Unfavorable outcome at 6 months	345 (39.3%)

hWFNS = Herniation World Federation of Neurosurgical Societies; ICP = Intracranial Pressure; ± SD = standard deviation; *the percentages were calculated upon the number of the cases with known values

### Relation between patients’ baseline characteristics and mTMT

**Fig. 2:**
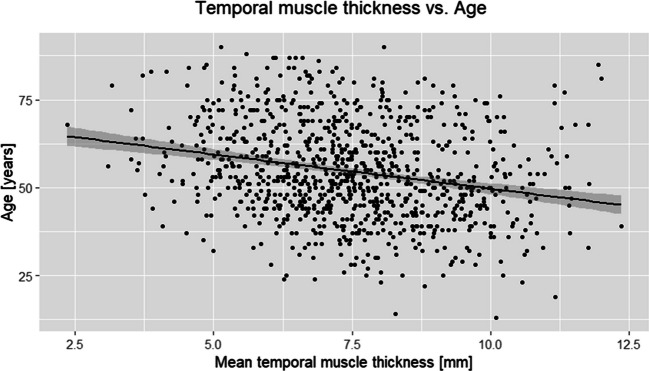
Scatterplot of patient populations age vs. TMT mean in millimeters

The mean mTMT value of the whole aSAH population was 7.49 mm ± 1.68 mm. Of the analyzed baseline characteristics, patients’ demographic characteristics (age, sex, and ethnicity) and some pre-existing conditions (obesity, uricemia, hypothyroidism) were significantly associated with mTMT values as illustrated in Table S1. The relationship between mTMT value and age (as a continuous variable) has been additionally visualized in Fig. 2.

### mTMT as predictor of adverse events

Furthermore, mTMT also showed significant correlations with the following adverse events in univariable analysis: ICP increase (7.7 ± 1.7 vs. 7.3 ± 1.7 mm for event present or absent, respectively [hereinafter], p = 0.003) and angiographic vasospasm (7.3 ± 1.7 vs. 7.6 ± 1.7 mm, p = 0.017). In contrast, there was no association between mTMT with aneurysm rebleeding (7.3 ± 1.9 vs. 7.5 ± 1.7 mm, p = 0.269), shunt dependency 7.5 ± 1.7 vs. 7.6 ± 1.7 mm, p = 0.451), DC (7.4 ± 1.7 vs. 7.5 ± 1.7 mm, p = 0.439), systemic infections (7.5 ± 1.8 vs. 7.4 ± 1.6 mm, p = 0.529), sepsis (7.3 ± 1.8 vs. 7.5 ± 1.7 mm, p = 0.588), as well as ACS (7.0 ± 1.3 vs. 7.5 ± 1.7 mm, p = 0.105) among others. In the final multivariable analysis (Table 2) mTMT was independently associated with the risk of ICP increase > 20 mmHg (aOR 1.67 per-mm-increase, 95% CI 1.06–1.29, p = 0.002) and angiographic vasospasm (aOR 0.87 per-mm-increase, 95% CI 0.78–0.97, p = 0.011).

**Table 2:** Multivariable analysis for associations between TMT and secondary study endpoints, which showed significant results in univariable analysis. The associations were adjusted for outcome and TMT-relevant confounders

Parameter	aOR	95% CI	p-value
*Secondary study endpoints*			
*ICP increase* > *20 mmHg needing treatment*			
mTMT	1.67	1.06–1.29	0.002
Age, per-year-increase	0.98	0.97–0.99	< 0.0001
Ethnicity (Caucasian)	1.36	0.62–3.01	0.446
Sex (female)	1.18	0.83–1.66	0.355
Obesity	1.02	0.56–1.87	0.946
Hypothyroidism	0.89	0.55–1.44	0.625
Hyperuricemia	0.71	0.26–1.94	0.500
Acute hydrocephalus	2.73	1.85–4.04	< 0.0001
hWFNS 5	13.32	5.57–31.82	< 0.0001
Fisher 3–4	2.80	1.64–4.76	< 0.0001
*Angiographic vasospasm*			
mTMT	0.87	0.78–0.97	0.011
Age, per-year-increase	0.96	0.94–0.97	< 0.0001
Ethnicity (Caucasian)	1.67	0.75–3.68	0.207
Sex (female)	1.54	1.04–2.27	0.032
Obesity	0.75	0.36–1.52	0.420
Hypothyroidism	0.96	0.57–1.62	0.881
Hyperuricemia	0.34	0.04–2.68	0.307
Acute Hydrocephalus	2.48	1.56–3.92	< 0.0001
hWFNS 5	0.31	0.15–0.64	0.002
Fisher 3–4	2.30	1.23–4.29	0.009
*In-hospital mortality*			
mTMT	0.86	0.75–0.99	0.035
Age, per-year-increase	1.05	1.03–1.07	< 0.0001
Ethnicity (Caucasian)	1.15	0.41–3.25	0.789
Sex (female)	0.70	0.43-1.12	0.135
Obesity	0.67	0.28–1.56	0.347
Hypothyroidism	0.88	0.46–1.69	0.709
Hyperuricemia	2.03	0.67–6.20	0.214
Acute hydrocephalus	1.58	0.85–2.94	0.145
hWFNS 5	9.13	4.91–16.96	< 0.0001
Fisher 3–4	3.59	1.05–12.32	0.042
ICP increase > 20 mmHg	5.53	3.40–9.00	< 0.0001
Angiographic vasospasm	1.06	0.65–1.74	0.820

mTMT = mean Temporal Muscle Thickness; hWFNS = Herniation World Federation of Neurosurgical Societies; ICP = Intracranial Pressure; aOR = adjusted Odds Ratio; CI = Confidence Interval

### mTMT as predictor of aSAH outcome

**Fig. 3:**
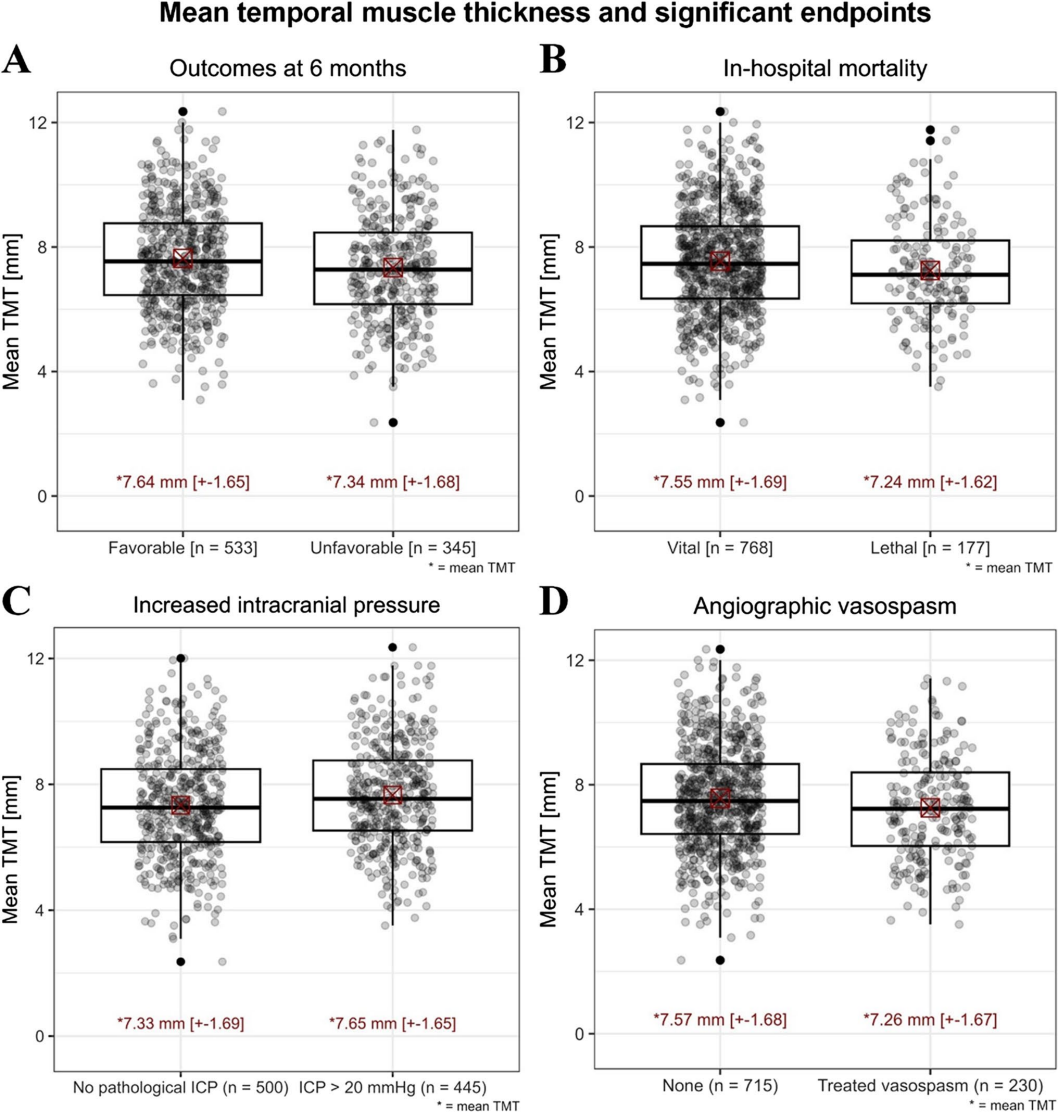
Illustration of mean ± SD temporal muscle thickness measurements in millimeters (mm) in relation to significant endpoints as there are (A) unfavorable outcome at 6 months, (B) in-hospital mortality, (C) increased ICP needing medical treatment and (D) angiographic vasospasm. Red square with cross stands for mTMT, black thick line for median. The results are divided into “occurrence” and “non-occurrence” of the respective endpoint. Abbreviations: TMT = Temporal Muscle Thickness; ICP = Intracranial Pressure; * = mean Temporal Muscle Thickness in mm; ± SD = Standard deviation in mm; n = Number of patients

Regarding the primary and secondary clinical outcomes and end points respectively, mTMT correlated in the univariable analysis with in-hospital mortality (7.2 ± 1.6 vs. 7.6 ± 1.7 mm, p = 0.031) and unfavorable outcome at 6 months as primary clinical outcome (7.3 ± 1.7 vs. 7.6 ± 1.7 mm, p = 0.009) but not with the risk of cerebral infarction (7.5 ± 1.7 vs. 7.5 ± 1.7 mm, p = 0.752). The multivariable analysis (Table 3) confirmed independent associations between the mTMT values with 6 months unfavorable outcome (aOR 0.86 per-mm-increase, 95% CI 0.78–0.96, p = 0.018) and (Table 2) in-hospital mortality (aOR 0.86 per-mm-increase, 95% CI 0.75–0.99, p = 0.035) as secondary endpoint. A summary of the correlation between mTMT and the significant endpoints, concerning adverse events as well as aSAH outcome, is displayed in Fig. 3.

**Table 3:** Multivariable analysis for associations between TMT and primary study endpoint which showed significant results in univariable analysis. The associations were adjusted for outcome and TMT-relevant confounders

Parameter	aOR	95% CI	p-value
*Primary study endpoint*			
*Unfavorable outcome at 6 months*			
mTMT	0.86	0.78–0.96	0.018
Age, per-year-increase	1.08	1.07–1.10	< 0.0001
Ethnicity (Caucasian)	1.38	0.58–3.25	0.466
Sex (female)	0.46	0.31–0.74	0.001
Obesity	1.27	0.58–2.81	0.551
Hypothyroidism	0.50	0.27–0.93	0.028
Hyperuricemia	0.92	0.28–3.00	0.891
Acute hydrocephalus	1.86	1.12–3.09	0.016
hWFNS 5	8.97	3.91–20.60	< 0.0001
Fisher 3–4	3.64	1.55–8.52	0.003
ICP increase > 20 mmHg	9.86	6.34–15.33	< 0.0001
Angiographic vasospasm	1.89	1.21–2.93	0.005

mTMT = mean Temporal Muscle Thickness; hWFNS = Herniation World Federation of Neurosurgical Societies; ICP = Intracranial Pressure; aOR = adjusted Odds Ratio; CI = Confidence Interval

## Discussion

In our study, we used a large European cohort including 945 patients to analyze the relationship between mTMT as a surrogate marker for sarcopenia and the clinical outcome of patients with aSAH. We identified a significant association between mTMT and both in-hospital mortality and unfavorable outcomes at 6 months, as well as with specific adverse events occurring during the acute phase of aSAH. Since these associations remained independent in multivariable analysis, further investigation into the underlying mechanisms linking sarcopenia with aSAH progression is warranted.

In the multivariable model analysis, we considered factors that were either significantly associated with the endpoints of the study and/or showed a relevant relationship with our variable of interest, mTMT. These included parameters such as patient-specific factors, initial clinical condition, severity of SAH, and early and secondary complications such as infections and vascular events. These variables have been consistently described in the literature as important predictors for the outcome of aSAH [7, 13].

Sarcopenia, as a condition of reduced muscle mass, predominantly among elderly people and critically ill patients, is associated with several adverse clinical outcomes, poor overall survival among various diseases as well as postoperative complications. Malnutrition, smoking, aging and diabetes mellitus among other comorbidities are associated with an increased risk of developing sarcopenia [48]. In addition, the effects of extended immobilization and critical illness on metabolic homeostasis also contribute to muscle loss [43]. Apart from a disturbance of the skeletal muscle homeostasis due to progressive age, other important pathophysiological mechanisms for the development of sarcopenia include a reduction in the size and number of type II muscle fibers accompanied by fat migration into the muscle, a reduction in satellite cell function, inflammation, insulin resistance, oxidative stress and dysfunction of neuromuscular junctions. These factors, in general, lead to a catabolic muscle metabolism, resulting in muscle wasting [2].

At the onset of a disease, sarcopenia represents an easily and early measurable prognostic parameter for the outcome of patients with various medical conditions. Considering critically ill patients with admission to intensive care unit, a meta-analysis based on retrospective cohort studies using CT scan predominantly at the beginning of medical history revealed a 2.28-fold mortality risk in patients with sarcopenia [49]. Hereby, sarcopenia was measured either by analyzing skeletal muscle area index, psoas or masseter muscle. Likewise, the meta-analysis of Shachar et al. showed, considering non-hematological solid tumors, that pre-treatment sarcopenia leads to poor overall survival (OS) [38]. Similar results regarding clinical outcome could be found in ischemic stroke patients [1] and patients undergoing cardiac surgery [30].

To predict the course of challenging diseases, the search for simple and easily accessible diagnostic tools is constantly being investigated. As a part of the skeletal muscle system, the temporalis muscle is also affected by these processes mentioned above and can be easily measured using CT scan. Thus, its thickness may reflect the level of overall muscle reduction in the context of sarcopenia. When talking about possible prognostic markers, TMT has already been examined several times in the literature regarding its significance for the outcome in various disease groups. For instance, Yan et al. found in 261 patients that a thicker TMT was associated with better OS in patients with high-grade glioma [47]. Similarly, in a recent systematic review and meta-analysis with 3283 glioblastoma patients, Sadhwani et al. confirmed a significant correlation between a thicker TMT and improved OS and progression-free survival [36]. There are also adverse reports on the link between TMT and OS after high-grade glioma surgery, as Wende et al. have reported [46]. Apart from various studies elucidating the relationship between low TMT and poor clinical outcome in high-grade glioma patients, there are also studies underlining its relevance in patients with secondary brain tumors [11], other tumor dignities, for example, oral cavity squamous cell carcinoma [29] as well as in those with aSAH, revealing partially inconsistent results. Katsuki et al. examined 127 patients and found a significant correlation between larger TMT and temporal muscle area (TMA) and a good clinical outcome [20]. The same research group also found a significant correlation between temporal muscle configuration and mRS in another population of 298 patients with aSAH who underwent endovascular aneurysm treatment and a population of 49 patients older than 75 years of age [19, 18]. Rodrigues et al. also found a link between TMT/TMA extent and poor functional outcome at discharge and six-month follow up in patients with intracranial aneurysms (199 ruptured, 162 unruptured) [35]. In contrast, Karadag et al. found no significant correlation between TMT/TMA of 478 European aSAH patients and the neurological outcome after six months defined by dichotomized mRS [17].

Regarding the mentioned studies, discrepancies in the findings can be attributed to several factors. First, it is noticeable that past studies used a distinct smaller patient cohort than ours, consisting of 49 to 478 patients, notwithstanding that homogeneity and geographical affiliation of the population may hinder direct comparison of findings. Additionally, highly specific and diverse subgroup analyses, inconsistent definitions, and unstandardized measuring protocols for TMT, for example, when it comes to diagnostic modalities, location of measurement as well as setting a cut-off value for when TMT is high or low, lead to a higher risk of incomparability across studies and may reduce the extended applicability of findings. These aspects, as well as heterogeneity in definitions of outcome parameters, therapeutical regimes for aSAH, and statistical analyses make it challenging to replicate or generalize results. These aspects emphasize the need for future standardized studies.

Our investigation on a large European cohort appears to reveal a relationship, which already have been reported for aSAH- [19, 20] and non aSAH-patients [12, 36]. We found a significant correlation between demographic factors such as sex, age, ethnicity, and mTMT. This possibly underlines the influence of baseline physiological characteristics on skeletal muscle health. Muscle mass naturally decreases when aging due to comorbidities and reduced physical activity, therefore, differences in TMT values across demographic groups may emerge from variations in lifestyle, genetic predispositions and metabolic health.

Moreover, our findings demonstrate that mTMT on admission correlates significantly with critical cerebral complications, such as increased ICP and angiographic vasospasm, but not with systemic complications.

Given the assumption of the protective effect of greater temporal muscle, it could be postulated that higher muscle mass may reflect better metabolic and systemic cardiovascular reserve, helping to maintain cerebral perfusion and thereby reduce the risk of cerebral edema [37]. However, patients in our study with higher mTMT values showed a higher probability of developing increased ICP that needed to be treated. An appropriate pathophysiological explanation remains to be investigated.

Angiographic vasospasm is a result of a complex interplay of neurovascular inflammation, dysregulated vascular tone and endothelial dysfunction [45]. Higher TMT values could possibly indicate better overall health, reducing the prevalence of endothelial dysfunction and improving vascular reactivity. The lack of significant association between TMT and systemic adverse events suggests that TMT may rather contribute as a local prognostic marker reflecting cerebral complications and pathophysiology. In turn, systemic complications and adverse events may be more influenced by immune response, infection control and organ function, which may overpower the impact of muscle mass.

Furthermore, we found a significant correlation between lower mTMT and unfavorable outcome at 6 months as well as in-hospital mortality. Higher TMT values probably indicate better physiological reserve, leading to more resilience and patients better withstanding the inflammatory and metabolic stress associated with aSAH, which direct to improved recovery trajectories. Additionally, lower TMT levels, as a surrogate for sarcopenia, indicate a catabolic state with probable chronic systemic inflammation leading to compromised recovery, which is associated with poorer outcomes in critically ill patients [49]. The lacking association between mTMT and cerebral infarction in our study may reflect the multifactorial nature of cerebral infarction.

In the current research phase, the clinical impact and the role of mTMT in clinical decision making still need to be elaborated and confirmed by further prospective research. Nevertheless, our results suggest that mTMT may serve as a reliable marker for outcome prediction in SAH patients and therefore may facilitate future therapy strategies and risk stratification.

For instance, mTMT, together with other established scores such as the Hunt & Hess, WFNS or Fisher score, could possibly help to identify patients at an early stage who are at increased risk of poor functional recovery or mortality. In the future, mTMT could potentially be integrated into artificial intelligence-supported decision-making algorithms as an additional radiologically measurable parameter to implement AI-based prognostic models and risk score systems.

Moreover, mTMT might be considered as part of intensive care monitoring to initiate individual treatment strategies such as early mobilization, optimized nutritional therapies, and to estimate the probability and susceptibility of secondary complications.

Furthermore, in the later phases of therapy, i.e. in early rehabilitation, it could potentially help to set up better nutritional medical concepts and muscle-building programs, to improve functional recovery and therefore long-term prognosis. These possible influences and considerations must be confirmed in the future by further studies and research.

## Limitations

The cross-sectional, retrospective, and monocentric study design is a major limitation of this study. In particular, TMT was only determined at a single timepoint (hospital admission) and used for further analysis. This provides a snapshot of the patients' muscle status, but it does not show changes in TMT over time, which would probably represent the clinical trajectory more accurately. To validate TMT as a reliable surrogate marker for sarcopenia and the clinical outcome of aSAH patients, larger, multicentric, longitudinal studies tracking TMT are necessary to provide more meaningful data and insights into how muscle wasting progresses in response to critical illness affects the clinical outcomes of patients. Moreover, multiple confounding factors like age, sex, comorbidities (including malignant and chronic diseases), nutritional status, preexisting frailty as well as genetics and ethnicity may influence the independent prognostic value of mTMT. In addition, we have not used a generally established threshold for TMT that can be used to indicate sarcopenia, because so far, it is not existent. This makes it difficult to compare the results of different studies, as well as the heterogeneity in measurement techniques also leads to a lack of comparability.

## Conclusion

Based on our study findings from a large, representative aSAH cohort, we observed a significant correlation between mTMT, a marker of sarcopenia, and clinical outcomes in aSAH patients. Lower mTMT values were associated with higher rates of in-hospital mortality and unfavorable outcomes at 6 months. Additionally, the patients' muscle mass condition may influence the risk of increased ICP and cerebral vasospasm following aSAH. Given its ease of measurement on CT scans and its strong association with functional outcomes and adverse events, mTMT could serve as a valuable prognostic marker to predict disease progression in aSAH patients. Further research is needed to validate its prognostic relevance and to better understand the underlying mechanisms involved.

## Supplementary Information

Below is the link to the electronic supplementary material.

Supplementary file1 (DOCX 14 KB)

## Data Availability

Data will be available upon reasonable request from the corresponding author.
