# Supplementary material for: Temporal muscle thickness as a feasible sarcopenia marker and outcome predictor after aneurysmal subarachnoid hemorrhage
Source: Acta Neurochir (Wien). 2025 May 29;167(1):157. doi: 10.1007/s00701-025-06562-z (PMC12122570; doi:10.1007/s00701-025-06562-z)
Supplement: Supplementary file 1 — Supplementary file1 (DOCX 14 KB) [file 701_2025_6562_MOESM1_ESM.docx]

Supplementary Data:

Table S1. Association between the mean TMT values and other study parameters

| **Parameter** | **TMT, mean (±SD) or CC:** | | **p-value** |
| --- | --- | --- | --- |
|  | **yes** | **no** |  |
| Age, years | CC: -0.239 | | <0.0001* |
| Sex, female | 7.1 (±1.6) | 8.3 (±1.6) | <0.0001* |
| Ethnicity, Caucasian | 7.5 (±1.7) | 8.1 (±1.4) | 0.026* |
| Arterial hypertension | 7.5 (±1.7) | 7.6 (±1.7) | 0.224 |
| Coronary heart disease | 7.3 (±1.6) | 7.5 (±1.7) | 0.153 |
| Diabetes mellitus | 7.7 (±1.9) | 7.5 (±1.7) | 0.389 |
| Peripheral arterial disease | 6.8 (±1.7) | 7.5 (±1.7) | 0.151 |
| Smoking | 7.5 (±1.6) | 7.5(±1.7) | 0.819 |
| Drug abuse | 6.9 (±1.8) | 7.5 (±1.7) | 0.149 |
| Obesity | 8.2 (±1.6) | 7.4 (±1.7) | 0.001* |
| Hypothyroidism | 7.6 (±1.7) | 7.0 (±1.5) | 0.001* |
| Hyperthyroidism | 7.7 (±2.1) | 7.5 (±1.7) | 0.743 |
| Hyperuricemia | 8.3 (±1.9) | 7.5 (±1.7) | 0.026* |
| Modified WFNS, Grade 5 | 7.5 (±1.5) | 7.5 (±1.7) | 0.790 |
| Fisher Grade 3-4 | 7.4 (±1.7) | 7.8 (±1.7) | 0.017* |
| Acute hydrocephalus | 7.4 (±1.7) | 7.7 (±1.6) | 0.015* |
| Aneurysm rebleeding | 7.3 (±1.9) | 7.5 (±1.7) | 0.269 |
| Shunt dependency | 7.5 (±1.7) | 7.6 (±1.7) | 0.451 |
| Epileptic seizures | 7.4 (±1.7) | 7.5 (±1.7) | 0.420 |
| Decompressive craniectomy | 7.4 (±1.7) | 7.5 (±1.7) | 0.439 |
| ICP increase requiring treatment | 7.7 (±1.7) | 7.3 (±1.7) | 0.003* |
| Angiographic vasospasm | 7.3 (±1.7) | 7.6 (±1.7) | 0.017* |
| Systemic infection | 7.5 (±1.8) | 7.4 (±1.6) | 0.529 |
| Sepsis | 7.3 (±1.8) | 7.5 (±1.7) | 0.588 |
| Acute coronary syndrome | 7.0 (±1.3) | 7.5 (±1.7) | 0.105 |
| Thromboembolic complications | 8.1 (±1.8) | 7.5 (±1.7) | 0.114 |
| Cerebral infarction | 7.5 (±1.7) | 7.5 (±1.7) | 0.829 |
| In hospital mortality | 7.2 (±1.6) | 7.6 (±1.7) | 0.020* |
| Unfavorable outcome at 6 months | 7.3 (±1.7) | 7.6 (±1.7) | 0.009* |

Abbreviations: CC = correlation coefficient; ICP = Intracranial Pressure; WFNS = World Federation of Neurosurgical
Societies; ±SD = standard deviation, * = significant p-value
